# A case report of concurrent embryonal rhabdomyosarcoma and diffuse large B-cell lymphoma in an adult without identifiable cancer predisposition

**DOI:** 10.1186/s40364-017-0086-7

**Published:** 2017-02-08

**Authors:** M. D. Mathias, M. V. Ortiz, H. Magnan, S. R. Ambati, E. K. Slotkin, A. J. Chou, M. F. Walsh, K. Offit, C. Moskowitz, A. Kentsis, L. H. Wexler

**Affiliations:** 1 Department of Pediatrics, New York, NY USA; 2 Department of Medicine, New York, NY USA; 3 Department of Leukemia, New York, NY USA; 4 Sloan Kettering Institute, Memorial Sloan Kettering Cancer Center, New York, NY USA; 5 Memorial Sloan Kettering Cancer Center, 1275 York Avenue, New York, NY 10065 USA

**Keywords:** Rhabdomyosarcoma, Diffuse large B cell lymphoma, Molecular analysis, Case report

## Abstract

**Background:**

Diffuse large B-cell lymphoma (DLBCL) is the most common form of non-Hodgkin lymphoma. Rhabdomyosarcoma, the most common soft tissue sarcoma of childhood. makes up less than 1% of solid malignancies in adults with around 400 new cases each year in the United States. They have not previously been reported concurrently.

**Case presentation:**

A 37 year old woman presented with painful enlarging leg mass. Biopsy of the mass was consistent with embryonal rhabdomyosarcoma. Staging imaging revealed a PET avid anterior mediastinal lymph node. Excisional biopsy of this mass was consistent with diffuse large B-cell lymphoma. Hybridization capture-based next-generation DNA sequencing did not reveal shared somatic tumor mutations. Germline analysis did not show identifiable aberrations of *TP53* or other heritable cancer susceptibility genes. She was treated with a personalized chemotherapy regimen combining features of R-CHOP and Children’s Oncology Group ARST 0331.

**Conclusions:**

This case illustrates a unique clinical entity successfully treated with a personalized chemotherapeutic regimen.

## Background

Rhabdomyosarcoma (RMS), the most common soft tissue sarcoma of childhood, is subdivided into three histopathologically distinct groups: embryonal, alveolar, and pleomorphic [1]. Embryonal RMS (ERMS) is the most common subtype. Alveolar RMS is characterized by the presence of a pathognomonic *FOXO1* gene rearrangement with *PAX3* or *PAX7*. Pleomorphic rhabdomyosarcoma is exceedingly rare in children and seen almost exclusively in adults. RMS makes up less than 1% of solid malignancies in adults with around 400 new cases each year in the United States [1, 2]. Therapy for patients with RMS is stratified based upon site-modified TNM staging and the extent of up-front surgical resection and typically involves multi-agent chemotherapy, and some combination of surgery with or without radiation therapy for local control.

**Fig. 1:**
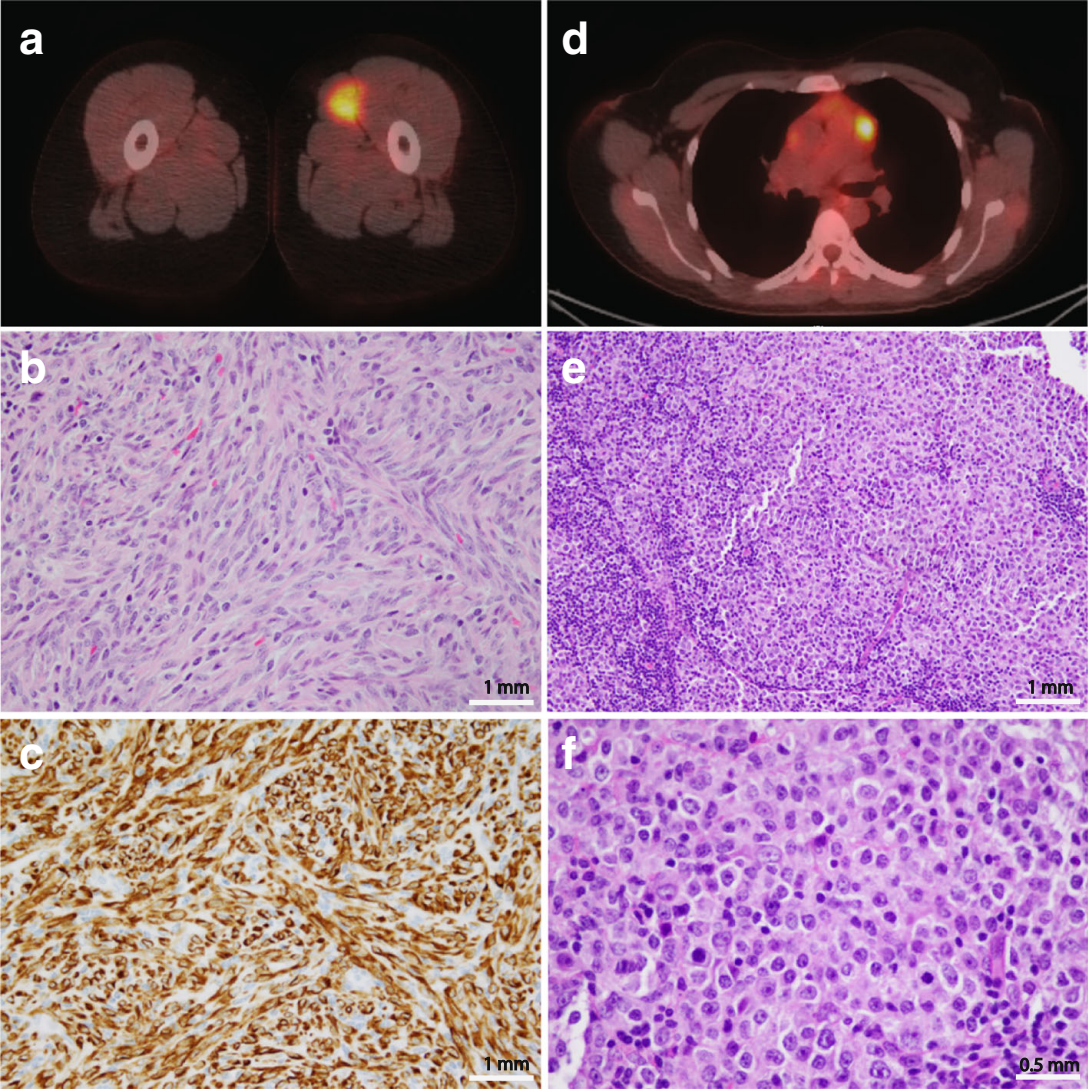
Key imaging and pathology results from the Rhabdomyosarcoma and Diffuse large B-cell lymphoma. **a** Axial view of the left thigh rhabdomyosarcoma demonstrating the lesion was FDG-avid on PET scan. **b** Morphology of rhabdomyosarcoma showing spindle cells in intersecting fascicles. (H&E, 200x) **c** Immunohistochemical stain for Desmin showing diffuse positivity supporting the diagnosis of rhabdomyosarcoma. **d** Axial view of the mediastinal diffuse large B-cell lymphoma demonstrating FDG-avidity on PET scan. **e** Morphology of lymph node showing involvement by diffuse large B-cell lymphoma. (H&E, 200x) **f** Higher power image of the lymphoma showing large lymphoid cells in sheets. (H&E, 400x)

While usually sporadic in appearance, RMS has been seen in patients with cancer-predisposition syndromes such as Li-Fraumeni syndrome, neurofibromatosis type I, Costello syndrome, Noonan syndrome, and Beckwith-Wiedemann syndrome [3]. There is also an increased risk of RMS in children with constitutional *DICER1* mutations [4]. Additionally, due to multimodal therapy with alkylating agents and radiation based therapy, these patients are also at risk of developing secondary malignancies. The last Childhood Cancer Survivor Study reported that 20% of patients with secondary malignancies were survivors of soft tissue sarcomas [5].

Diffuse large B-cell lymphoma (DLBCL) is the most common form of non-Hodgkin lymphoma. Heterogeneous in presentation and molecular pathology, it is characterized by aberrant proliferation of mature B-cells in nodal or extranodal sites [6]. Therapy is based on extent of disease and presence or absence of ‘B symptoms’ and includes a multi-agent chemotherapy with targeted therapy including anti-CD20 antibodies. Genome wide association studies have shown independent single-nucleotide polymorphisms (SNPs) correlate with an increased risk of DLBCL [7, 8]. We report a woman with no significant prior medical history who was diagnosed with simultaneous ERMS and DLBCL. To our knowledge, the synchronous occurrence of these two tumors has not previously been reported in the medical literature. Next generation sequencing did not reveal a pervasive somatic or germline mutation that might link these tumors. Testing for a germline mutation consistent with a cancer predisposition syndrome failed to reveal identifiable abnormality of *TP53* or any of the other genes associated with an increased risk of soft tissue sarcomas, in general, or rhabdomyosarcoma in particular.

## Case presentation

A 37-year old woman with no pertinent past medical history presented with a firm non-tender lump in her left proximal thigh. The mass was mildly painful and enlarged over one year. This patient had a family tree consisting of 2 generations above and 1 generation below compiled during her visit with the Clinical Genetics team. The family cancer history was notable for a paternal grandfather with prostate cancer at age 80, a maternal uncle with prostate cancer at age 72, and a maternal grandmother with melanoma at age 60. There were no cancers at an early age, sarcomas, leukemias, lymphomas, nor malignancies pathognomonic for inherited predisposition syndromes.). Approximately one month prior to diagnosis, she was hospitalized for management of pneumonia; imaging studies were not performed and she was treated with antibiotics and a weeklong pulse of glucocorticosteroids. Following ultrasound-guided fine needle aspiration showing a spindle cell neoplasm, a MRI demonstrated an enhancing intra-muscular mass measuring 4.9 cm in maximal diameter. She underwent en bloc resection of the mass with pathology consistent with high-grade spindle cell rhabdomyosarcoma with high mitotic rate, negative margins, and negative local lymph nodes. During staging imaging, she was found to have a hypermetabolic 1.4 cm left anterior mediastinal perivascular thoracic lymph node on baseline PET scan (SUV = 6.6). As the presence of a distant metastasis would increase her stage from Stage 2 to Stage 4, with a resultant significant change in both her therapy and prognosis, she underwent excision of this mediastinal mass which revealed a distinct primary tumor, DLBCL. Given the simultaneous occurrence of two rare and pathophysiologically distinct malignancies, each tumor was sequenced using MSK-IMPACT, a hybridization capture-based next-generation DNA sequencing assay of exons of 410 genes recurrently mutated in solid tumors [9] (Fig. 1). Her rhabdomyosarcoma was negative for *FOXO1* gene rearrangement and found to have a somatic alteration in *NF1*. Her DLBCL had multiple somatic mutations including *ATM*, *B2M*, *FAT1*, *HGF*, *MYCN*, *PIM1*, various mutations in the same allele of exon 2 of *SOCS1*, and *SOX17* (Table 1). MSK-IMPACT testing did not reveal any germline mutations in the targeted gene panel. No copy number alterations were identified. Although the ERMS was of sufficient tumor purity, the DLBCL had low tumor content. Mean overall coverage (sequencing depth) in the ERMS, DLBCL, and germline samples were 606X, 623X, and 261X, respectively. Given the consequences of missing a Li-Fraumeni syndrome diagnosis, additional testing was performed on the *TP53* coding sequence (exons 2–11), including bidirectional splice junction sequencing, as well as deletion and duplication analysis to evaluate for larger chromosomal rearrangements were negative for any germline aberrations.

**Table 1:** Somatic tumor gene alterations within each tumor

Tumor	Somatic Tumor Gene Alterations
Rhabdomyosarcoma of the Left Thigh	1. *NF1* (NM_001042492) exon26 p.G1128fs (c.3382_3383delGG)
Diffuse Large B-Cell Lymphoma of the Anterior Mediastinum	1. *ATM* (NM_000051) exon58 p.S2860del (c.8578_8580delTCT)
	2. *B2M* (NM_004048) exon1 p.L15fs (c.43_44delCT)
	3. *FAT1* (NM_005245) exon25 p.T4225M (c.12674C > T)
	4. *HGF* (NM_000601) exon17 p.G660W (c.1978G > T)
	5. *MYCN* (NM_005378) exon3 p.R393H (c.1178G > A)
	6. *PIM1* (NM_002648) exon4 p.L143M (c.427C > A)
	7. *SOCS1* (NM_003745) exon2 p.H87fs (c.258_268delGCACGGGGCGC)
	8. *SOCS1* (NM_003745) exon2 p.R92G (c.274C > G)
	9. *SOCS1* (NM_003745) exon2 p.S143N (c.428G > A)
	10. *SOCS1* (NM_003745) exon2 p.A184T (c.550G > A)
	11. *SOX317* (NM_022454) exon1 p.P49L (c.146C > T)

Based on the absence of distant metastases and the favorable histology of her tumor, her RMS was classified as “low-risk” (Stage 2, Group I) and her mediastinal DLBCL was classified as Stage 1. Consequently, she was treated with an individualized regimen that incorporated the basic design of the Children’s Oncology Group (COG) low-risk rhabdomyosarcoma study, ARST 0331, with R-CHOP. The low risk arm of ARST 0331 is composed of 24 weeks of chemotherapy with vincristine, dactinomycin, and cyclophosphamide plus/minus radiation. R-CHOP consists of rituximab, cyclophosphamide, doxorubicin, vincristine, and prednisone. The patients individualized regimen is detailed in Table 2. Because severe neuropathy in adults tends to limit the tolerability of intensively-dosed vincristine as typically given on pediatric chemotherapeutic regimens, her individualized regimen was modified to replace 6 vincristine doses with 2 additional cyclophosphamide doses. Four doses of dactinomycin were substituted with doxorubicin to achieve the desired anthracycline dose for R-CHOP. She received pulses of prednisone every 5 days and 6 total doses of Rituximab. Her treatment course was complicated by severe constipation and jaw pain secondary to vincristine, granulocyte colony stimulating factor associated bone pain, and mild mucositis during her expected periods of neutropenia. These toxicities did not lead to any delays in treatment. She is currently more than a year and a half off therapy in complete remission from both primary cancers, undergoing close monitoring for both malignancies.

**Table 2:** Individualized treatment regimen

Week	Cyclophosphamide 1.2 gm/m^2^ IV x1	Dactinomycin 0.045 mg/kg IV x1	Doxorubicin 50 mg/kg IV x1	Prednisone 100 mg daily x5 days	Rituximab 375 m/m^2^ IV x1	Vincristine 1.5 mg/m^2^ IV x1	Disease surveillance scans
1	X	X		X	X	X	
2						X	
3						X	
4	X		X	X	X	X	
5						X	
6						X	
7	X		X	X	X		
8							
9							
10	X		X	X	X		
11							
12							X
13	X		X	X	X		
14							
15							
16	X	X		X	X		
17						X	
18						X	
19		X		X	X	X	
20						X	
21						X	
22		X		X	X	X	
23							
24							X

## Discussion and Conclusions

We report an adult woman with no prior medical history nor familial cancer history who was diagnosed with synchronous RMS and DLBCL. Genomic analyses did not identify unifying somatic nor predisposing germline mutations. Although there is a prior report of a 64 year old man with concurrent RMS and Hodgkin lymphoma, to our knowledge this is the first case of RMS and any non-Hodgkin lymphoma [10].

Two distinct primary tumors without prior chemotherapy or radiation exposures or known underlying cancer predisposition is a rare entity that is poorly described. The simultaneous existence of two primary tumors presents a major therapeutic challenge, requiring the tailoring of a customized regimen that provides adequate therapy directed at each tumor without incurring intolerable toxicities. Cyclophosphamide, doxorubicin, vincristine, and prednisone are active against both RMS and DLBCL [11, 12]. It is likely that this overlapping chemotherapeutic sensitivity allowed the patient to tolerate the combined regimens of the COG RMS protocol with standard DLBCL therapy, R-CHOP.

The patient received a week pulse of steroids for pneumonia one month before she was diagnosed with RMS. This may have partially treated her DLBCL, causing her mediastinal mass to be less extensive than if untreated. A week-long steroid pre-phase is routinely utilized in the treatment of elderly patients with low performance status as a means of reducing initial treatment toxicities [13].

Despite genomic analyses, no unifying molecular aberration was identified in both of our patient’s two cancers. Archer et al. argued that the SEER-9 database provides population based evidence that subgroups of patients with rhabdomyosarcoma have constitutional cancer predisposition [14]. At this point, further studies are required to delineate whether our patient had the unfortunate coincidence of two unrelated synchronous tumors or indeed represents part of a yet to be fully characterized cohort of patients with constitutional or inherited risk for rhabdomyosarcoma and other cancers.

## Abbreviations

COG: Children’s oncology group
DLBCL: Diffuse large B-cell lymphoma
ERMS: Embryonal RMS
RMS: Rhabdomyosarcoma
SNPs: single-nucleotide polymorphisms
